# New Hypothesis for Cause of Epidemic among Native Americans, New England, 1616–1619

**DOI:** 10.3201/edi1602.090276

**Published:** 2010-02

**Authors:** John S. Marr, John T. Cathey

**Affiliations:** Virginia Commonwealth University School of Medicine, Richmond, Virginia, USA (J.S. Marr); King Faisal Specialist Hospital and Research Centre, Riyadh, Saudi Arabia (J.T. Cathey)

**Keywords:** Native Americans, Weil disease, leptospirosis, bacteria, viruses, yellow fever, smallpox, plague, trichinosis, historical review

## Abstract

In the years before English settlers established the Plymouth colony (1616–1619), most Native Americans living on the southeastern coast of present-day Massachusetts died from a mysterious disease. Classic explanations have included yellow fever, smallpox, and plague. Chickenpox and trichinosis are among more recent proposals. We suggest an additional candidate: leptospirosis complicated by Weil syndrome. Rodent reservoirs from European ships infected indigenous reservoirs and contaminated land and fresh water. Local ecology and high-risk quotidian practices of the native population favored exposure and were not shared by Europeans. Reduction of the population may have been incremental, episodic, and continuous; local customs continuously exposed this population to hyperendemic leptospiral infection over months or years, and only a fraction survived. Previous proposals do not adequately account for signature signs (epistaxis, jaundice) and do not consider customs that may have been instrumental to the near annihilation of Native Americans, which facilitated successful colonization of the Massachusetts Bay area.

## Abstract 2

This epidemic may have been leptospirosis complicated by Weil syndrome.

Retrospective studies have inherent, sometimes insurmountable, biases, but speculation on past events by historians and anthropologists is commonplace and offers grist for future studies. We offer an alternative hypothesis for the cause of an epidemic among Native Americans in the years immediately before the arrival of the Pilgrims in Massachusetts. During 1616–1619, many persons died of a disease that presumably spared nearby European fishermen and traders (*1*). The more severe manifestations were fever, headache, epistaxis, jaundice, and skin lesions. Speculations as to the cause have included plague, yellow fever, and smallpox (*2*–*7*), as well as influenza, chickenpox, typhus, typhoid fever, trichinosis, cerebrospinal meningitis, and syndemic infection of hepatitis B virus (HBV) and hepatitis D virus (HDV) (Table 1) (*6**–**11*). We propose another disease: leptospirosis, accompanied by Weil syndrome. With its more severe manifestations, this syndrome is consistent with available clinical information, the nidality of *Leptospira* organisms, the introduction of rodent reservoirs, and the presence of favorable ecologic niches. Practices of the local population placed it repeatedly in high-risk exposures to epidemic and hyperendemic environments.

**Table 1:** Summary of published interpretations suggesting or discounting possible causes of an epidemic among Native Americans, New England, 1616–1619*

Cause	Suggested	Discounted
Yellow fever	Webster (*2*)	Cook (*1*), Williams (*3*), Carter (*4*), Bratton (*6*)
Plague	Williams (*3*)	Carter (*4*), Hoornbeek (*5*), Bratton (*6*), Crosby (*9*)
Influenza	Carter (*4*)	
Smallpox	Bratton (*6*), Holmes (*7*)	Cook (*1*), Webster (*2*), Williams (*3*), Hoornbeek (*5*)
Chickenpox	Hoornbeek (*5*), Cronon (*10*)	Bratton (*6*)
Typhus	Lescarbot (*11*)	Williams (*3*), Bratton (*6*)
HBV/HDV	Speiss and Speiss (*8*)	
Leptosopirosis	This study	

*HBV, hepatitis B virus; HDV, hepatitis D virus.

## Epidemiology

The limited information available notes the following clinical manifestations of the illness: headache and fever with visible signs of epistaxis and jaundice. Mode of transmission was not known. Weather and seasonality are unknown, although tree ring data suggest greater than average rainfall in eastern Massachusetts during 1615–1625 (*12*). The duration of the epidemic (or epidemics) reportedly ranged from 3 to 6 years. Estimated death rates (which lack reliable numerator and denominator data) range from one third of the local population to as high as 90% (*1*,*13*). The Patuxet (Plimouth) Native American village was severely depopulated (*14*). Referring to conditions along the Newfoundland and Maine coasts, where some believe the epidemic may have originated, Pierre Biard, a Jesuit missionary, noted: “They [the Indians] are astonished and often complain that since the French mingle and carry on trade with them, they are dying fast, and the population is thinning out” (*15*). In New England, Smith noted “three plagues in three years successively neere two hundred miles along the coast” of southern Massachusetts to Cape Cod and inland for 15 miles (*16*). Bennett suggested a 50–60-mile interior extension, which corresponds to the area of native corn horticulture (*17*).

**Figure 1:**
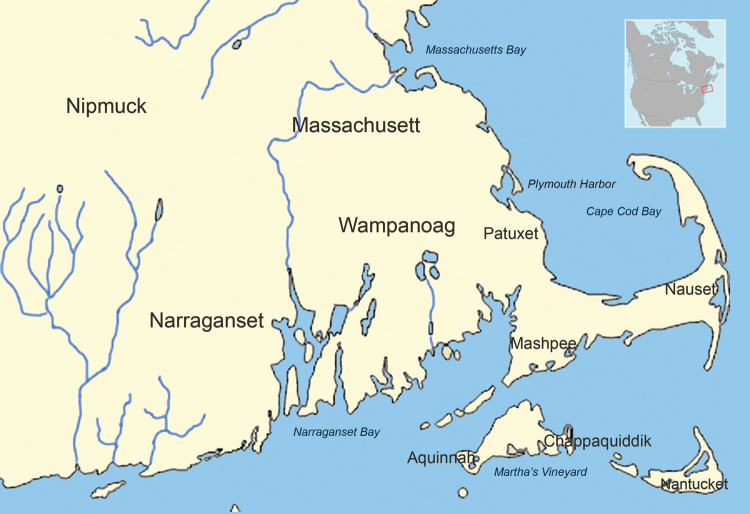
Native American tribes of southeastern Massachusetts in ≈1620.

**Figure 2:**
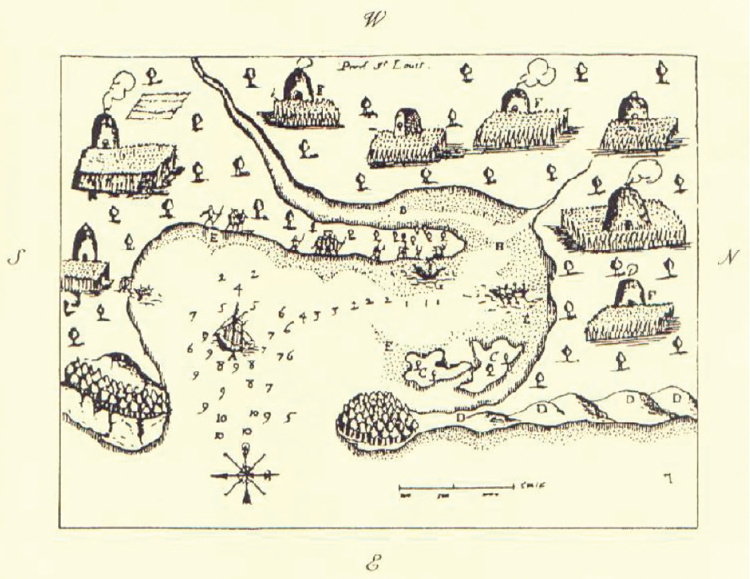
Plymouth, Massachusetts, harbor showing extensive Native American settlement (a sketch by Samuel de Champlain from his voyage of 1606).

By 1616, several subtribes of the Wampanoag (Pokanoket) Nation were living between the present-day borders of eastern Rhode Island and southeastern Maine (Figure 1). The Patuxet village was localized to an area in and around Plymouth harbor (Figure 2). Demographers and historians disagree about the total size of the Wampanoag Nation, but Salisbury considers an estimate of 21,000–24,000 as “not unrealistic for this region” (*13*). Gookin also estimated 3,000 men living in Massachusetts before the epidemic (*18*), which when extrapolated for family size is consistent with Salisbury’s overall estimate. Salisbury estimated that the size of the Patuxet tribe before the epidemic was 2,000.

No estimates are available of the number of Portuguese, Breton, and Bristol fishermen; Basque whalers; French fur traders; or English codders who had established a presence on the North Atlantic coast since the early sixteenth century (*10*). In 1578, an observer noted 100 Spanish sails, 20–30 Basque whalers, ≈150 French and Breton fishing ships, and 50 English sails along the coast of Newfoundland (*19*). English traders and fishermen had daily contact with indigenous persons but lived on ships or in segregated enclaves on land where salt-dried codfish stations (favored by the English) were built along Massachusetts Bay.

## Ecology

Indigenous ecology was cataloged in 1604 when hundreds of coastal plants, trees, and animals (but not “vermine”) were described (*20*). Before 1620, there were no peridomiciliary animals except for small dogs and mice (*10*), although other rodents (e.g., squirrels) were common. Precolonization and postcolonization English written accounts do not mention rats, the numbers of which may have been influenced by the presence of cats, but aboard ships rats must have been common. An earlier explorer noted “Tant qu’on eut des cuirs on ne s’avisa point de faire la guerre aux rats…” (“As long as there is a cargo of skins, it makes no sense to kill the rats.”) (*11*). The black rat (*Rattus rattus*) was common in coastal England at the time (yet to be displaced by the brown rat [*R*. *norvegicus*] nearly 100 years later) (*21*); the black rat and mice were universal companions on ships and must have established themselves early on the coastal mainland, seeking harborage in and around Native American households. Once established, rats and mice would become chronic carriers of disease agents, contaminating water and soil and infecting other commensal rodents (e.g., the local mouse *Peromyscus leucopus*) and other mammals. Fresh and stored food items such as maize, beans, squash, pumpkin, roots, nuts, berries, meat, fish, and shellfish, were also susceptible to leptospiral contamination.

## Previous Explanations

One hundred years ago, Williams collected all known information about the epidemic in an article that included 23 primary references, 22 of which contained eyewitness accounts or reports (*3*). He concluded that the disease may have been bubonic plague and supported his proposal by noting that there were abundant fleas in Indian dwellings, survivors had sores suggestive of buboes, and plague was endemic in London during 1606–1611. Eleven of his 23 primary sources disagreed, as did Carter, who without further elaboration stated that he thought the epidemic was influenza (*4*). Despite allusions to icterus, Williams discounted yellow fever (as did Carter); he also dismissed other febrile illnesses with jaundice, yet he cited Gookin from 1674: “I have discoursed with old Indians, who were then youths, who say that the bodies all over were exceedingly yellow, describing it by a yellow garment they showed me, both before they died and afterwards.” Trumbull, another eyewitness, noted that the Indian word for the disease meant “a bad yellowing” (*3*). A recent analysis interpreted it as caused by a confluent form of smallpox (*6*). Clinical and epidemiologic information about classical explanations and some of the more recent suggestions are summarized in Table 2.

**Table 2:** Factors related to some of the postulated causes of an epidemic among Native Americans, New England, 1616–1619*

Factor	Yellow fever	Plague	Influenza	Smallpox	Chickenpox	Typhus	HBV/HDV	Leptospirosis
Characteristic signs and symptoms								
Headache/fever	Yes	Yes	Yes	Yes	Yes	Yes	Yes	Yes
Jaundice	Yes	No	No	No	No	No	Yes	Yes
Hemorrhages	Yes	Yes	No	Yes	No	Yes	No	Yes
Skin lesions†	Bruises	Buboes	No	Pustules	Rash	Rash	No	Rash
Epidemiologic								
High attack rate	Yes	Yes	Yes	Yes‡	Yes	Yes	Yes	Yes
High death rate	Yes	Yes	No	Yes	No	Yes	Yes	Yes
Endemic in Europe	No	Yes	Yes	Yes	Yes	Yes	No	Yes
Suitable arthropod vector	No	Yes	NA	NA	NA	Yes	NA	NA
Suitable reservoir host	No	No	Yes	Yes	Yes	Yes	Yes	Yes
Native susceptibility	Yes	Yes	Yes	Yes	Yes	Yes	Yes	Yes
European susceptibility	Yes	Yes	Yes	No	No	Yes	Yes	Yes

*HBV, hepatitis B virus; HDV, hepatitis D virus; NA, not applicable. †Sign mentioned by only 1 person (Thomas Dermer) and possibly referred to another unrelated disease outbreak. ‡Native Americans only.

## Discussion

The causes of most historical epidemics may never be proven. The new science of paleomicrobiology may provide some answers, but the question will remain about whether a person died of a specific disease or with the disease. However, even when proper evidence is limited, this limitation should not dissuade speculation about the causes of ancient afflictions. Our hypothesis is not meant to be a definite answer but a heuristic for others to criticize and explore. Alfred Crosby, one of America’s foremost medical historians, coined the term “virgin soil epidemics” to describe immunologically unexposed populations exposed to Old World diseases and cited the 1616–1619 epidemic as an example (*9*). He also proposed that environmental and behavioral factors were equally important (*22*). The Massachusetts epidemic supports this observation, and evidence may indicate that “genetic weakness” was not as important as the intimate and repeated exposure to an infectious agent among the Indians not shared by Europeans.

All previously proposed explanations for the epidemic are consistent with an Old World importation into a susceptible population (except for Webster’s, who thought yellow fever was of autochthonous origin). Despite its manifestation and subsequent visitations along coastal America in later years, yellow fever is not a plausible explanation given the routes of the trans-Atlantic slave trade at the time. Transportation of the disease, its vector, and human cargo from Africa to the New World was limited to the Caribbean and Central and South America; little evidence exists that any ships visited the New England coast after disembarking slaves (*23*). Alternative arthropod-borne and other non-arthropod–borne viral hemorrhagic fevers are even less plausible candidates.

Clinical descriptions of other proposed diseases (plague, chickenpox, typhus, typhoid fever, and meningitis) are largely inconsistent with the syndrome described and were dismissed by Bratton. Citing Oliver Wendell Holmes, Sr. (*7*), Bratton concluded that the disease was smallpox, explaining that the confluent form of pustular smallpox might mimic jaundice (*6*). In 1799, Webster had discounted smallpox because “the Indians, who were perfectly acquainted with the disease [smallpox] after the English arrived, always gave a very different account of it...” (*2*). Two diseases not mentioned by Bratton (trichinosis and HBV/HDV infections) are also unlikely. Pigs were absent in the New World, and the finding of a single pig bone in an undated midden makes a most unlikely explanation for the epidemic. Syndemic HBV/HDV infection presupposes aboriginal HBV carriage, HDV importation, and (in the opinion of Speiss and Speiss) an enteric mode of transmission (*8*).

In 1886, Adolf Weil originally described a constellation of signs and symptoms that is now eponymic for Weil syndrome (his first patient experienced nasenbluten [nosebleed] on the second day of illness) (*24*). Inada and Ido identified the causative organism 30 years later (*25*). Subsequent studies have demonstrated that rodents have high rates of leptospiral carriage and shedding (*26*). Severe (icteric) leptospirosis was also known as infectious jaundice, epidemic jaundice, and icto-hemorrhagic fever (*27*). Early outbreaks in the United States were recorded by Neill, including a Union Civil War Surgeon General’s report of a large number of “hepatic and haematic disorders” estimated to have affected >71,000 troops during the War (*28*).

In 1965, Heath et al. summarized the history of leptospirosis in the United States, analyzing 483 cases reported during 1949–1961 (*29*,*30*). Twenty-five percent were caused by *L*. serovar Icterohemorhagiae. Today, *L*. Icteroheamorrhagiae and other serovars (Canicola, Autumnalis, Hebdomidis, Australis, and Pomona) are endemic in the United States, and isolated instances within the United States continue to be reported (*31*). More recent reports from the Centers for Disease Control and Prevention (*32*,*33*) and ProMED mail (*34*) demonstrate that leptospirosis is a worldwide, reemerging infection with identifiable risk factors, including immersion in fresh water, exposure to contaminated soil, and antecedent heavy rains (*35*,*36*). Unlike hookworm disease, another Old World soil-borne disease that established itself in the more hospitable American South, leptospirosis is a more cosmopolitan fellow traveler and is still recognized as a zoonosis in New England.

Contemporary medical texts conflate signs, symptoms, and death rates of mild leptospiral infection with Weil syndrome, relying on more recent citations in which the nature of exposure, duration, and responsible *Leptospira* spp. are often not known. Interventional measures (removal from known sources, prompt diagnosis and treatment, and early prevention and control measures) may have decreased overall case-fatality rates and limited the extent of the outbreaks. Nosebleed is rarely mentioned in the recent literature, but “hemorrhages, starting with epistaxis” are noted in a 1944 text on tropical diseases, which also cites high death rates (32% in Europe and 48% in Japan) (*27*). These surprisingly high death rates in early Japanese reports were attributed to repeated intimate exposure to contaminated water by barefooted mine workers and rice farmers.

Unlike the European experience, epidemics in Japan were rare, and endemic exposures were more common (*27*). A recent population-based seroepidemiologic study found leptospiral seropositivity rates of 28% in an annually flooded area of the Amazon basin (*37*). *Leptospira* spp. were found to cause seasonal outbreaks of a mysterious disease (tentatively named Andaman hemorrhagic fever) during periods of rice paddy sowing and harvesting in the late 1980s on the Andaman Islands in the Indian Ocean (*38*). Subsequent studies found that leptospiral seroposivity was as high as 62.5% (among agricultural workers) in the Andaman Islands and that the case-fatality rate was 42.9% among hospitalized patients with severe leptospirosis and pulmonary symptoms.

**Figure 3:**
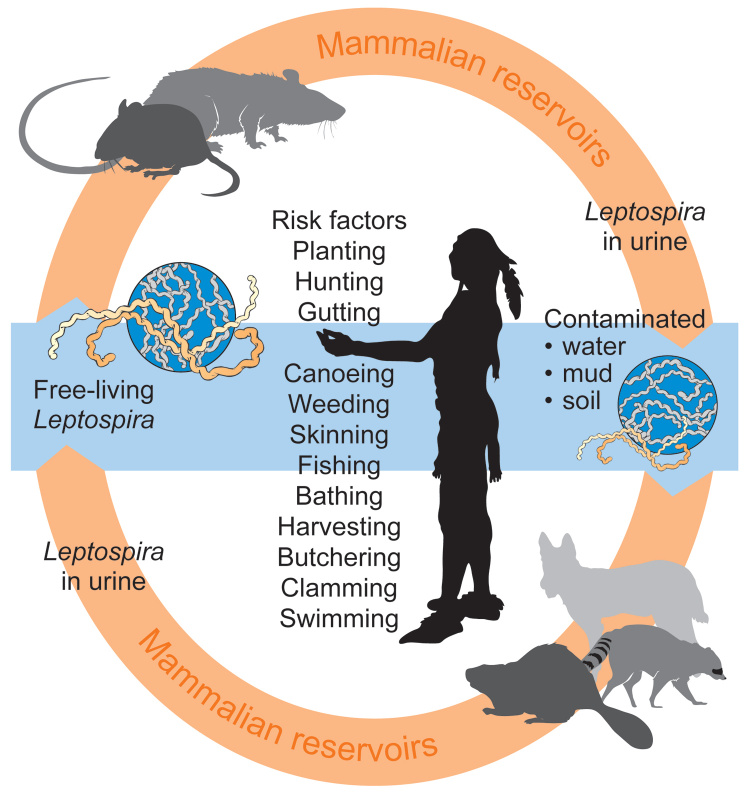
Depiction of how a leptospiral life cycle might have affected Native Americans in New England (1616-1619) due to behavioral and environmental risk factors.

Endemicity and subsequent high case-fatality rates, similar to those reported from Japan, are consistent with a leptospiral etiology for the 1616–1619 epidemic. The Patuxets may not have associated sickness with their environment or traditional ways of living and may have attributed their affliction to many causes, but not to countless exposures and reexposures to the agent. Sporadic, focal mini-epidemics may have played out and coalesced into what was construed as a single “plague” by outside observers. Except for more severe cases of liver failure, the most common cause of death for leptospirosis (renal or respiratory insufficiency) would have not been recognized. The Indian lifestyle, which included constant exposure to rodents and their excreta on land and in water, exposed them to the leptospiral life cycle (Figure 3) (*39*,*40*). Bare feet were common in and around houses. Although a rare portal of entry, mucosal exposure may have occurred from ingestion of corn buried in the ground in rodent-accessible baskets and from rodent-contaminated foods in wigwams (weetas). Dermal abrasions offered cutaneous portals of entry. Attendance of the ill and burial of the dead (including those who died from Weil syndrome) would have attracted others who shared local food, water, and camp grounds. It was common practice for entire families to enter sweat lodges followed by immediate immersion in cooling streams and ponds; sweat lodges were considered vivifiers and cure-alls for illnesses, a practice that may have reexposed the already ill to contaminated water. Once the spirochete established its presence in numerous foci, it survived for months in water, mud, and moist soil and caused infection in additional mammalian reservoirs. A reduction in the populace may have been incremental, episodic, and continuous; daily needs and customs may have exposed the Indians to leptospirosis over many months or years, with only a small fraction of the population eventually surviving. Suggestions that the disease persisted among the Indians after 1619 (perhaps through 1630) support the premise of endemic nidality and selective Indian vulnerability. The fate of nearby European cod fishermen is unknown, but they did not share most of the Indians’ risk factors. Boots would have limited transmission from fresh water exposures, bathing was not a common practice, and work in a saline environment may have curtailed transmission. An occasional case of febrile illness on board ship would have been attributed to many other causes. Disease and death may have occurred among the fishermen but are not recorded.

The exact duration and extent of the epidemic(s) will never be known, but our suggestion offers an alternative explanation. Persistent leptospiral exposures resulted in more severe cases of Weil syndrome and jaundice, a sign that would have been reported by observers; the cause of death from other (anicteric) leptospiral infection would not have been recognized. Our proposal is consistent with the historical clinical descriptions, estimated death rates, importation and distribution of its reservoir host, inoculation of the agent in multiple suitable nidalities, spread to other mammalian reservoirs, hyperendemicity, ecologic factors favoring repeated exposure and transmission, and known high-risk activities of the indigenous population.

The name Squanto has entered American history and folklore as the one of the last of the Patuxets who assisted the Pilgrims in 1620. He was one of the few survivors of an epidemic that was crucial to the success of the Plymouth and Massachusetts Bay colonies because remaining Indians had little capacity to resist the new settlers. Two years later, after having fever and a nosebleed, Squanto died of what was then referred to as “the Indean disease.”
